# Retraction Note: Stimulator of IFN genes mediates neuroinflammatory injury by suppressing AMPK signal in experimental subarachnoid hemorrhage

**DOI:** 10.1186/s12974-025-03570-9

**Published:** 2025-10-16

**Authors:** Yucong Peng, Jianfeng Zhuang, Guangyu Ying, Hanhai Zeng, Hang Zhou, Yang Cao, Huaijun Chen, Chaoran Xu, Xiongjie Fu, Hangzhe Xu, Jianru Li, Shenglong Cao, Jingyin Chen, Chi Gu, Feng Yan, Gao Chen

**Affiliations:** Department of Neurosurgery, The Second Affiliated Hospital of Zhejiang University, School of Medicine, Jiefang Road 88th, Hangzhou, 310000 China

**Retraction Note: J Neuroinflamm 17, 165 (2020)**

**https://doi.org/10.1186/s12974-020-01830-4**

The Editors-in-Chief have retracted this article. After publication, concerns were raised regarding some of the images presented in this article, specifically,

1. Figure 3 C: Panels sham and SAH appear to overlap;

2. Figure 4B: Panels sham and SAH + C-176 appear to overlap;

3. Figure 6 A: Panels sham CA3 and SAH + C176 CA3 appear to overlap.

An investigation by the Publisher also found that the western blots in this article appear similar to those in unrelated articles [1, 2]. The Editors-in-chief have therefore lost confidence in the presented data.

Authors did not respond to correspondence from the Publisher about this retraction.
